# Supplementary material for: Higher fibrinogen-to-albumin ratio is associated with the severity of toxin associated acute kidney injury in high-altitude (3650 m) population: A retrospective analysis
Source: PLoS One. 2026 Jan 12;21(1):e0330776. doi: 10.1371/journal.pone.0330776 (PMC12795372; doi:10.1371/journal.pone.0330776)
Supplement: S1 Table — Table 1. Comparison of demographic and laboratory characteristics between poisoned AKI patients (n = 8) defined without regard to urine output criterion (AKI-2) and non-AKI-2 patients admitted to the hospital. Note: AKI, acute kidney injury; SBP, systolic blood pressure; DBP, diastolic blood pressure; bpm, beats per minute; AST, aspartate aminotransferase; ALT, alanine aminotransferase. If the mean/ average or the highest or lowest value during the disease was not specified, the values were considered to be the laboratory result at onset. Table 2. Results of Spearman bivariate correlation analysis for the highest serum creatinine concentration during the illness. (DOCX) [file pone.0330776.s001.docx]

**Supplementary materials:**

Table 1. Comparison of demographic and laboratory characteristics between poisoned AKI patients (n=8) defined without regard to urine output criterion (AKI-2) and non-AKI-2 patients admitted to the hospital.

| mean±SD/ median(interquartile range) | AKI-2 (n=8) | Non-AKI- 2(n=57) | Statistics(T value/ Mann‒Whitney Z value/ χ^2^） | Sig.（two-tailed） |
| --- | --- | --- | --- | --- |
| Age, years | 51.5(30.6,62.5) | 36.0(29.1,46.9) | 1.468 | 0.142 |
| Male, n(%) | 6(75.0%) | 19(33.3%) | 3.536 | 0.060 |
| Length of stay, days | 12.5(5.0, 15.5) | 4.0(3.0,7.0) | 2.438 | 0.015 |
| Proteinuria positive or suspected, n(%) | 5(71.4%) (n=7) | 10(19.2%) (n=52) | 6.326 | 0.012 |
| Serum Urea, mmol/L | 8.8(6.6,9.6) (n=7) | 4.2(3.2,5.6) (n=52) | 2.954 | 0.002 |
| Serum Creatinine, µmol/L | 113.9(93.7, 222.0) | 62.2(50.0, 75.8) (n=53) | 3.996 | <0.001 |
| Highest urea during disease, mmol/L | 9.1(5.3, 9.6) | 5.0(3.8,6.2) | 2.586 | 0.010 |
| Highest creatinine during disease, µmol/L | 113.9(93.7,222.0) | 64.0(52.0,78.7) | 3.934 | <0.001 |
| Blood myoglobin, ng/mL | 357.5(104.0,1200.0) (n=7) | 66.3(30.4,209.9) (n=34) | 1.907 | 0.058 |
| SBP, mmHg | 125.3±22.9 | 110.9±17.2 | 2.127 | 0.037 |
| DBP, mmHg | 85.8±19.7 | 70.0±13.7 | 2.868 | 0.006 |
| Heart rate, bpm | 103.0±33.7 | 79.2±16.5 | 1.963 | 0.088 |
| Blood lymphocyte percentage, % | 8.0(5.2,12.0) (n=7) | 18.4(10.8,26.2) (n=53) | -2.429 | 0.013 |
| White blood cell, *10^9^/L | 14.3(11.9,14.7) (n=7) | 9.0(6.4,12.9) (n=55) | 2.213 | 0.025 |
| Neutrophil percentage, % | 85.5±7.1 | 76.6±11.9 (n=53) | 2.051 | 0.045 |
| Neutrophils/lymphocyte ratio | 10.9(7.4,20.1) (n=7) | 4.3(2.6, 7.2) (n=51) | 2.423 | 0.014 |
| Fibrinogen, g/L | 3.7(2.5,4.3) (n=6) | 2.6(2.2, 3.1) (n=54) | 2.008 | 0.043 |
| FAR: Fibrinogen-albumin ratio, % | 10.2(7.2, 14.3) (n=6) | 6.4(5.4,8.1) (n=53) | 2.132 | 0.032 |
| Plasma D-dimer, µg/mL | 5.4(0.5,15.6) (n=6) | 0.5(0.3,1.0) (n=48) | 2.147 | 0.030 |
| Fibrinogen degrading product, µg/mL | 5.1(3.0, 47.5) (n=7) | 2.1(1.2,3.1) (n=55) | 2.080 | 0.037 |
| AST, U/L | 103.0(46.5,424.0) (n=7) | 24.0(18.5,42.0) (n=51) | 2.436 | 0.013 |
| ALT, U/L | 114.0(38.0,351.0) (n=7) | 23.0(16.0,48.0) (n=50) | 2.104 | 0.033 |
| Average direct bilirubin, mmol/L | 6.6(4.6,18.1) (n=7) | 5.0(3.5,7.0) (n=55) | 1.624 | 0.105 |
| Average total bilirubin, mmol/L | 26.0(15.8,34.1) (n=7) | 16.1(11.3, 23.9) (n=55) | 1.346 | 0.186 |
| Blood glucose, mmol/L | 8.3(5.8,10.3) | 5.8(5.3,7.8) (n=50) | 1.624 | 0.108 |
| Average SBP, mmHg | 123.5±19.8 | 110.8±12.3 | 2.534 | 0.014 |
| Average DBP, mmHg | 83.6±12.3 | 71.4±10.1 | 3.121 | 0.003 |
| Average heart beats, bpm | 92.4±14.5 | 78.4±10.4 | 2.641 | 0.030 |

Note: AKI, acute kidney injury; SBP, systolic blood pressure; DBP, diastolic blood pressure; bpm, beats per minute; AST, aspartate aminotransferase; ALT, alanine aminotransferase. If the mean/ average or the highest or lowest value during the disease was not specified, the values were considered to be the laboratory result at onset.

Table 2. Results of Spearman bivariate correlation analysis for the highest serum creatinine concentration during the illness.

|  | N | correlation coefficient | Sig. (two-tailed) |
| --- | --- | --- | --- |
| Mean serum urea, mmol/L | 65 | .485^**^ | <0.001 |
| Male | 65 | .582^**^ | <0.001 |
| Average hemoglobin, g/L | 63 | .522^**^ | <0.001 |
| Serum uric acid, µmol/L | 44 | .509^**^ | <0.001 |
| Blood myoglobin, ng/ml | 41 | .371^*^ | 0.017 |
| Serum phosphate, mmol/L | 55 | .307^*^ | 0.022 |
| FAR: Fibrinogen-albumin ratio | 59 | 0.208 | 0.114 |
| Average altitude, meters | 65 | -0.228 | 0.068 |
| Age, years | 65 | 0.191 | 0.127 |
| Total bilirubin, mmol/L | 62 | 0.184 | 0.152 |
| Serum calcium, mmol/L | 54 | 0.187 | 0.175 |
| Proteinuria | 65 | 0.163 | 0.196 |
| Diastolic blood pressure, mmHg | 64 | 0.158 | 0.213 |
| Systolic blood pressure, mmHg | 64 | 0.154 | 0.225 |
| White blood cells, *10^9^/L | 62 | 0.142 | 0.272 |
| Average heart beats, bpm | 65 | -0.131 | 0.298 |
| Comorbidities (0, none; 1, chronic disease including mental disorder; 2, infection) | 65 | 0.124 | 0.326 |
| Serum potassium, mmol/L | 65 | 0.122 | 0.333 |
| Poisoning types (1, medicine; 2, food; 3, organophosphate) | 65 | -0.053 | 0.677 |
| Highest urine output during the illness, mL/day | 55 | 0.124 | 0.367 |
| Neutrophil/lymphocyte ratio | 58 | 0.035 | 0.794 |
| Length of stay, days | 65 | 0.033 | 0.796 |
| Duration from poisoning to the clinic, hours | 62 | 0.018 | 0.891 |
