# Supplementary material for: Higher fibrinogen-to-albumin ratio is associated with the severity of toxin associated acute kidney injury in high-altitude (3650 m) population: A retrospective analysis
Source: PLoS One. 2026 Jan 12;21(1):e0330776. doi: 10.1371/journal.pone.0330776 (PMC12795372; doi:10.1371/journal.pone.0330776)
Supplement: Attachment — Submitted filename: response to reviewers.docx [file pone.0330776.s004.docx]

PONE-D-24-26186

Higher fibrinogen-to-albumin ratio predicts the severity of toxic kidney injury in patients of the plateau area—a retrospective analysis

**Dear editors and reviewers:**

We sincerely appreciate your insightful feedback and constructive suggestions regarding the manuscript. We have replied every question or advice step by step according to the order as the following.

PLOS ONE

Dear Dr. Yang,

Thank you for submitting your manuscript to PLOS ONE. After careful consideration, we feel that it has merit but does not fully meet PLOS ONE’s publication criteria as it currently stands. Therefore, we invite you to submit a revised version of the manuscript that addresses the points raised during the review process.

Please submit your revised manuscript by May 24 2025 11:59PM. If you will need more time than this to complete your revisions, please reply to this message or contact the journal office at [plosone@plos.org](mailto:plosone@plos.org). When you're ready to submit your revision, log on to https://www.editorialmanager.com/pone/ and select the 'Submissions Needing Revision' folder to locate your manuscript file.

Please include the following items when submitting your revised manuscript:

- A rebuttal letter that responds to each point raised by the academic editor and reviewer(s). You should upload this letter as a separate file labeled 'Response to Reviewers'.
- A marked-up copy of your manuscript that highlights changes made to the original version. You should upload this as a separate file labeled 'Revised Manuscript with Track Changes'.
- An unmarked version of your revised paper without tracked changes. You should upload this as a separate file labeled 'Manuscript'.

**Reply**: OK. They have been uploaded.

If you would like to make changes to your financial disclosure, please include your updated statement in your cover letter. Guidelines for resubmitting your figure files are available below the reviewer comments at the end of this letter.

**Reply**: Thanks a lot. We have included the updated statement in the cover letter. The figure was submitted as requirement.

If applicable, we recommend that you deposit your laboratory protocols in protocols.io to enhance the reproducibility of your results. Protocols.io assigns your protocol its own identifier (DOI) so that it can be cited independently in the future. For instructions see: [https://journals.plos.org/plosone/s/submission-guidelines#loc-laboratory-protocols](https://track.editorialmanager.com/CL0/https:%2F%2Fjournals.plos.org%2Fplosone%2Fs%2Fsubmission-guidelines%23loc-laboratory-protocols/1/010f01961ae2ba98-346298be-1c6c-4362-8ecc-cf9c30c61804-000000/S5h5fD8F5RBg1hz0OeWZzCaRebOFXLw_V_LMsS1MavA=206).

Additionally, PLOS ONE offers an option for publishing peer-reviewed Lab Protocol articles, which describe protocols hosted on protocols.io. Read more information on sharing protocols at [https://plos.org/protocols?utm_medium=editorial-email&utm_source=authorletters&utm_campaign=protocols](https://track.editorialmanager.com/CL0/https:%2F%2Fplos.org%2Fprotocols%3Futm_medium=editorial-email%26utm_source=authorletters%26utm_campaign=protocols/1/010f01961ae2ba98-346298be-1c6c-4362-8ecc-cf9c30c61804-000000/oM_juS7H4krQddTAXMSt0DGzDNApwk6LQoLA5k2m8FU=206).

**Reply:** Since the laboratory tests involved were all routine examinations, we didn’t deposit it in protocols.io. For convenience, we uploaded the dataset with our revised manuscript.

We look forward to receiving your revised manuscript.

Kind regards,

Mohammad Barary
Academic Editor
PLOS ONE

Journal Requirements:

When submitting your revision, we need you to address these additional requirements.

1. Please ensure that your manuscript meets PLOS ONE's style requirements, including those for file naming. The PLOS ONE style templates can be found at

[https://journals.plos.org/plosone/s/file?id=wjVg/PLOSOne_formatting_sample_main_body.pdf](https://track.editorialmanager.com/CL0/https:%2F%2Fjournals.plos.org%2Fplosone%2Fs%2Ffile%3Fid=wjVg%2FPLOSOne_formatting_sample_main_body.pdf/1/010f01961ae2ba98-346298be-1c6c-4362-8ecc-cf9c30c61804-000000/G24UWpFtgk0LfZ8Qzn1JbTxsOvIkf1FO9eNGR2kZXNY=206) and

[https://journals.plos.org/plosone/s/file?id=ba62/PLOSOne_formatting_sample_title_authors_affiliations.pdf](https://track.editorialmanager.com/CL0/https:%2F%2Fjournals.plos.org%2Fplosone%2Fs%2Ffile%3Fid=ba62%2FPLOSOne_formatting_sample_title_authors_affiliations.pdf/1/010f01961ae2ba98-346298be-1c6c-4362-8ecc-cf9c30c61804-000000/ABbwFbfGbb1rzUwLxXd4yuklCuet87_61wErZgnu3FM=206)

**Reply**: Yes, I have revised the manuscript as the style requirements.

2. Thank you for stating the following financial disclosure:

“This work was supported in part by a research Initiation Fund Sponsored by Peking University Third Hospital for Excellent Returned Overseas Scholars (BYSYLXHG2019007).”

Please state what role the funders took in the study.  If the funders had no role, please state: "The funders had no role in study design, data collection and analysis, decision to publish, or preparation of the manuscript."

If this statement is not correct you must amend it as needed.

Please include this amended Role of Funder statement in your cover letter; we will change the online submission form on your behalf.

**Reply**: Thank your suggestion. It has been stated in the cover letter including the other statements not be acceptable in the manuscript.

3. Your ethics statement should only appear in the Methods section of your manuscript. If your ethics statement is written in any section besides the Methods, please move it to the Methods section and delete it from any other section. Please ensure that your ethics statement is included in your manuscript, as the ethics statement entered into the online submission form will not be published alongside your manuscript.

**Reply**: Thanks. The ethic statement has been moved to the Methods section.

4. In this instance it seems there may be acceptable restrictions in place that prevent the public sharing of your minimal data. However, in line with our goal of ensuring long-term data availability to all interested researchers, PLOS’ Data Policy states that authors cannot be the sole named individuals responsible for ensuring data access ([http://journals.plos.org/plosone/s/data-availability#loc-acceptable-data-sharing-methods](http://track.editorialmanager.com/CL0/http:%2F%2Fjournals.plos.org%2Fplosone%2Fs%2Fdata-availability%23loc-acceptable-data-sharing-methods/1/010f01961ae2ba98-346298be-1c6c-4362-8ecc-cf9c30c61804-000000/f1-FyV-ub1J92LpIzK5UWH70kueCesXqRM0enIwdWgs=206)).

Data requests to a non-author institutional point of contact, such as a data access or ethics committee, helps guarantee long term stability and availability of data. Providing interested researchers with a durable point of contact ensures data will be accessible even if an author changes email addresses, institutions, or becomes unavailable to answer requests.

Before we proceed with your manuscript, please also provide non-author contact information (phone/email/hyperlink) for a data access committee, ethics committee, or other institutional body to which data requests may be sent. If no institutional body is available to respond to requests for your minimal data, please consider if there any institutional representatives who did not collaborate in the study, and are not listed as authors on the manuscript, who would be able to hold the data and respond to external requests for data access? If so, please provide their contact information (i.e., email address). Please also provide details on how you will ensure persistent or long-term data storage and availability.

**Reply**: Following consultations with the relevant hospital departments, we are able to provide the minimal data.

5. Please include captions for your Supporting Information files at the end of your manuscript, and update any in-text citations to match accordingly. Please see our Supporting Information guidelines for more information: [http://journals.plos.org/plosone/s/supporting-information](http://track.editorialmanager.com/CL0/http:%2F%2Fjournals.plos.org%2Fplosone%2Fs%2Fsupporting-information/1/010f01961ae2ba98-346298be-1c6c-4362-8ecc-cf9c30c61804-000000/WnhL-ac-qgFD9LW8Rnyd5k2shPsRbuAqLx1OMZ2BF4w=206).

**Reply**: OK. I have revised as asked.

Reviewers' comments:

Reviewer's Responses to Questions

**Comments to the Author**

1. Is the manuscript technically sound, and do the data support the conclusions?

The manuscript must describe a technically sound piece of scientific research with data that supports the conclusions. Experiments must have been conducted rigorously, with appropriate controls, replication, and sample sizes. The conclusions must be drawn appropriately based on the data presented.

Reviewer #1: Yes

Reviewer #2: Partly

**Reply:** Thanks for your support.

2. Has the statistical analysis been performed appropriately and rigorously?

Reviewer #1: Yes

Reviewer #2: Yes

**Reply:** Thanks for your support.

3. Have the authors made all data underlying the findings in their manuscript fully available?

The [PLOS Data policy](http://track.editorialmanager.com/CL0/http:%2F%2Fwww.plosone.org%2Fstatic%2Fpolicies.action%23sharing/1/010f01961ae2ba98-346298be-1c6c-4362-8ecc-cf9c30c61804-000000/QrBGs1aVCyirW8_wpcm5tgxbW94tJf1lQnvJrng-r9s=206) requires authors to make all data underlying the findings described in their manuscript fully available without restriction, with rare exception (please refer to the Data Availability Statement in the manuscript PDF file). The data should be provided as part of the manuscript or its supporting information, or deposited to a public repository. For example, in addition to summary statistics, the data points behind means, medians and variance measures should be available. If there are restrictions on publicly sharing data—e.g. participant privacy or use of data from a third party—those must be specified.

Reviewer #1: Yes

Reviewer #2: No

**Reply**: Following consultations with the relevant hospital departments, we are able to provide the minimal data to made all data underlying the findings in their manuscript fully available.

4. Is the manuscript presented in an intelligible fashion and written in standard English?

PLOS ONE does not copyedit accepted manuscripts, so the language in submitted articles must be clear, correct, and unambiguous. Any typographical or grammatical errors should be corrected at revision, so please note any specific errors here.

Reviewer #1: Yes

Reviewer #2: Yes

**Reply:** Thanks for your support.

5. Review Comments to the Author

Please use the space provided to explain your answers to the questions above. You may also include additional comments for the author, including concerns about dual publication, research ethics, or publication ethics. (Please upload your review as an attachment if it exceeds 20,000 characters)

Reviewer #1: Reviewer comments and suggestions regarding the manuscript entitled “Higher fibrinogen-to-albumin ratio predicts the severity of toxic kidney injury in patients of the plateau area—a retrospective analysis” with the manuscript number “PONE-D-24-26186.” 
This  retrospective  analysis  investigates  the  role  of  the  fibrinogen-to-albumin  ratio  (FAR)  in predicting the severity of toxic kidney injury among poisoned patients in high-altitude (plateau) regions.  Applying  KDIGO  acute  kidney  injury  criteria  and  performing  multivariate  linear regression analyses on clinical and laboratory data, the study finds that a higher FAR—along with elevated serum uric acid and phosphate levels—is significantly associated with worse renal function (as measured by serum creatinine). These findings suggest that as reflected by FAR, micro-inflammation could serve as a prognostic biomarker in this unique clinical setting.  
The  paper  can  have  an  essential contribution  to  its  field,  but  authors  should  address  some significant considerations: 
I.  Title: 
The informative title accurately reflects the study’s focus on FAR and its predictive value for 
toxic kidney injury in the plateau area.

**Reply**: Thanks.

II.  Abstract: 
The abstract summarizes the background, methods, key results, and conclusions. It efficiently communicates the central finding that FAR and other factors correlate with increased serum creatinine levels and, hence, more severe kidney injury.

**Reply**: Thanks.

III.  Introduction: 
The introduction sets the context by highlighting the variability in poisoning-related kidney 
injury and the limited use of AKI criteria in high-altitude regions. It justifies exploring novel 
biomarkers like FAR in this specific setting. **A more detailed discussion on the physiological 
relevance of FAR in inflammation and kidney injury could further enhance this section.**

**Reply:** The physiological relevance of FAR in inflammation and kidney injury was discussed in the second paragraph of the discussion section. As requested, I added the following sentence about the physiological relationship of FAR in inflammation and kidney injury in the introduction section:

“Emerging evidence suggests that fibrinogen-to-albumin ratio (FAR) [9], as novel inflammatory biomarkers, is significantly associated with AKI, potentially mediated through their reflection of microinflammatory states, oxidative stress pathways, and endothelial dysfunction implicated in both glomerular and tubular damage”.

IV.  Materials and Methods: 
The methods section is comprehensive, detailing the retrospective design, inclusion criteria, data sources (electronic medical records), and statistical techniques (e.g., independent t-tests, chi-square tests, Spearman correlation, and multivariate regression). The description of the 
analytical  approach  for  determining  risk  factors  is  clear.  **Additional details on handling missing data would help understand the study’s robustness.**

**Reply: Thanks for your advice.** For variables with missing data, we report the amount how many patients had this information. Ex: For missing data in urine volume or proteinuria, we provide the description with the number how many patients had this information in table 1 as 1150.0 (750.0, 1670.0) ml/d (n= 42) or 9 (18.8%) (n= 48) in non-AKI group (n= 53), which suggest that in the 53 patients without AKI, only 42 patients had urine volume information and 48 patients had information of whether the patient had proteinuria.

To make full use of the data with missing values such as total bilirubin and hemoglobin etc, we used the averaged value of that at disease onset and that at discharge in the comparison analysis. Pairwise deletion was utilized in correlation assessments. In the multivariate linear regression analysis, we designated the highest SCr value rather than the value at onset or discharge as the dependent variable.

**We added the above additional details on handling missing data in the paragraph about the statistics in Method section.**

**In addition, we added the shortcoming in the limitation section in lines 250**: “Potential biases may be introduced by **missing data** in the laboratory results, toxin dose and comorbidity etc.”

V.  Results: 
The results are well-organized and present the demographic and clinical characteristics of the 
study population.

**Reply**: Thanks.

VI.  Discussion: 
The discussion integrates the findings with existing literature on poisoning, inflammation, 
and kidney injury. The authors elaborate on the potential mechanisms linking FAR to renal 
dysfunction and underscore the importance of microinflammation in toxic AKI.

**Reply：**Thanks for your understanding and support.

VII.  Limitation: 
Limitations, such as the retrospective design and small sample size, are acknowledged. **A 
more in-depth exploration of potential confounders and regional factors (e.g., high-altitude physiology) would strengthen the discussion.**

**Reply:** Thanks for your suggestion. So, we made the following revision:

1) Even though no statistically significant difference/ relationship, we added a row of the average altitude in table 1 and S2 table.

2) We added two paragraphs in discussion section before the limitation part about the physiology or pathophysiology effect relating to high altitude.

“Exposure to high altitude may induce activation of hypoxia-inducible factors and eliciting reactive oxygen species. Hypoxia-inducible factors promote the expression of erythropoietin, vascular endothelial growth factor etc, promoting erythropoiesis and angiogenesis. However, we didn’t find any difference in the hemoglobin levels between AKI and non-AKI group. Excessive reactive oxygen species production leads to oxidative stress and scavenging of vascular nitric oxide bioavailability, which leads to vascular endothelial dysfunction [31]. Erythropoiesis as well as platelet adhesiveness, endothelial dysfunction, oxygen stress, and factors promoting hemoconcentration contributed to elevated risk of thrombogenesis in high altitude residents, especially above 3500 m [31.32]. Increased vascular permeability was postulated to reduce albumin levels due to leakage into interstitial spaces. Meanwhile, increased plasma levels of fibrinogen reported [10, 11] suggests higher FAR may be induced by high-altitude hypobaric hypoxia.

Hypoxia caused macrophage accumulation in the renal tissue and renal fibroblasts activation, increasing the deposition of extracellular matrix. Maladjustments to hypoxia may result in a variety of conditions including hypertension, kidney injury, etc [12]. Intermittent hypobaric hypoxia exposure was reported to induce oxidative stress along with kidney and liver injury, unusual expression of the uric acid synthesis / excretion regulator and inflammatory response, resulting in hyperuricemia [34]. All these may contribute to kidney injury related to high altitude exposure.”

3) We add the content in limitation section as the following: “Third, it is worth noticing that these findings should be interpreted with caution due to our relatively small sample size which may have risk of overfitting and **other unidentified variables. such as data about hypoxia or duration of high-altitude residence prior to assessment.**”

VIII.  Conclusion: 
The  conclusions  succinctly  summarize  the  study’s  findings  and  their  implications.  The 
manuscript effectively suggests that FAR could be a practical, easily obtainable biomarker 
for assessing kidney injury severity in poisoned patients. The call for further prospective 
studies  to  validate  these  findings  is  appropriate  and  highlights  the  need  for  continued research. 

**Reply：**Thanks for your positive remark.

IX.  References: 
The comprehensive and current reference list supports the study’s context and findings. 

**Reply：**Thanks for your positive remark.

X.  Declaration: 
Declarations regarding funding, ethics, and competing interests are appropriately stated and align with the journal’s requirements. 

**Reply：**Thanks for your positive remark.

XI.  Tables: 
The tables are detailed and well-structured, providing clear comparisons between patients 
with and without AKI. They effectively highlight statistically significant differences (e.g., 
higher urea and creatinine in the AKI group) and the correlations between key biomarkers. 
Supplementary  materials  further  support  the  findings.  The  regression  model  results  are 
clearly stated, with coefficients and confidence intervals enhancing the credibility of the analysis.

**Reply：**Thanks for your positive remark.

Reviewer #2: Dear Authors,
Thank you for submitting your manuscript, "Higher fibrinogen-to-albumin ratio predicts the severity of toxic kidney injury in patients of the plateau area—a retrospective analysis," to PLOS ONE. Your study provides valuable insights into the prognostic role of the fibrinogen-to-albumin ratio (FAR) in toxic kidney injury within a unique high-altitude population. The retrospective design and focus on plateau physiology offer novel contributions to the literature. However, several methodological and analytical aspects require clarification to strengthen the manuscript. Below, I provide detailed recommendations to enhance the scientific rigor, clarity, and clinical relevance of your work.
Major Comments
1. Title and Abstract
• Title: Replace "toxic kidney injury" with "acute kidney injury" (AKI) to align with KDIGO criteria. Specify the altitude range (e.g., "high-altitude [3650 m] population") to better characterize your study population.

**Reply:** Thanks for your advice. I have revised the title as you suggested.

• Abstract:
o De-emphasize the neutrophil-to-lymphocyte ratio (NLR), as it was not significant in final models, and instead highlight the clinical implications of FAR (e.g., risk stratification or therapeutic decision-making).

**Reply:** I have corrected the abstract as the following.

“Recent evidence suggests that the fibrinogen-to-albumin ratio is significantly associated with the incidence and severity of acute kidney injury, highlighting its potential clinical utility for risk stratification and prognostication in high-risk populations. We investigated the clinical characteristics and risk factors of toxic kidney injury in highland areas using the acute kidney injury criteria defined by KDIGO, with focus on the significance of the fibrinogen-to-albumin ratio.”

o Correct "fulfil" to "fulfill" for consistency with American English.

**Reply:** Thank you. I have corrected as advised.

o Clarify the clinical significance of the FAR coefficient (−0.078) in practical terms (e.g., how this translates to changes in creatinine levels).

**Reply:** We added the following at the end of the results of the abstract: “The study demonstrated a dose-dependent association between elevated fibrinogen-to-albumin ratio and increased serum creatinine levels”.

As for the practical terms of the relationship between FAR and the highest serum creatine concentration, we prepared the following table to illustrate the specific relationship, in which we suppose the uric acid concentration was 302.9umol/L and the serum phosphate level was 1.06mmol/L, the mean values in this study.

We calculated the highest serum creatinine concentration with the formula in the manuscript, from which we can see the highest serum creatinine concentration increases with FAR. The median (interquartile) of FAR values was 6.6 (5.4, 8.6) % or 0.066 (0.054, 0.086) in this study.

For example, we suppose FAR is 6.6% (=0.066), then the reciprocal of the highest SCr concentration (µmol/L) was 0.032 - 0.002* (302.9 (µmol/L) /100)- 0.005* 1.06 (mmol/L) -0.078* 0.066 = 0.015494. Thereby, the highest SCr concentration was 1 / 0.015494 = 64.541umol/L.

| FAR value (%) | The highest SCR concentration, umol/l |
| --- | --- |
| 1 | 50.347 |
| 2 | 52.405 |
| 3 | 54.639 |
| 4 | 57.071 |
| 5 | 59.730 |
| 5.4 | 60.864 |
| 6.4 | 63.898 |
| 6.6 | 64.541 |
| 7.7 | 68.325 |
| 8.5 | 71.367 |
| 10.9 | 143.021 |

2. Introduction
• Rationale: Strengthen the link between FAR and toxic AKI by citing specific mechanisms (e.g., fibrinogen’s role in microvascular thrombosis or hypoalbuminemia’s contribution to oxidative stress).

**Reply**：The following revision was made as advised.

“Emerging evidence suggests that fibrinogen-to-albumin ratio (FAR) [9], as novel inflammatory biomarkers, is significantly associated with AKI, potentially mediated through their reflection of microinflammatory states, oxidative stress pathways, and endothelial dysfunction implicated in both glomerular and tubular damage.”

• Plateau relevance: Expand on how high-altitude physiology (e.g., chronic hypoxia) might uniquely influence FAR or AKI risk.

**Reply**: We added the contents in introduction as the following: “Chronic exposure to hypoxia at high altitudes was reported to increase plasma fibrinogen level [10, 11], which suggest it might elevate FAR. Hypoxia promotes fibroblast activation in renal tissue, together with inflammatory cell recruitment and tubular epithelial cell damage, leads to renal tubule interstitial fibrosis [12]”. Also, in the discussion, we added the mechanism of high-altitude physiology -FAR- AKI relationship.

• Hypothesis: State explicitly (e.g., "We hypothesized that FAR would predict AKI severity independent of traditional inflammatory markers").

**Reply**: Thank you for your advice. We have added this sentence in the introduction.

3. Methods
• Participants:
o Justify the exclusion of patients lacking both urine tests and renal function data, as this may introduce selection bias.

**Reply**: Thank for your suggestion. We can’t judge whether he/she had AKI without creatinine results according to the KDIGO criterion. The patients should have at least one time of serum creatinine examination. All patients except one had data of serum creatinine. Neither this patient had urine test nor urine volume record, so he was excluded from analysis.

We revised this in the method part as the following:

“Patients who did not have urine volume record and renal function data during the illness (n=1) were excluded.”

o Provide more details on the altitude range (3650 m) and its physiological implications (e.g., effects on inflammatory markers or renal hemodynamics).

Reply: Thank for your suggestion. We added two paragraphs in the discussion about the details of altitude and physiological implications.

• AKI definition: Clarify how baseline creatinine was determined for patients without prior measurements (e.g., back-calculation, population median).

Reply: Most patients had performed two times of serum creatinine examinations (at onset and at discharge). For those with only one test of creatinine, we made diagnosis of AKI with reference to the criteria of urine volume and clinical diagnosis without adopting the methods of back-calculation or population median etc.

We have stated in the method part as the following: “An increase in serum creatinine (SCr) concentration from baseline can be substituted as a decrease from the baseline during follow-up if there are no prior tests. A urine output less than 400 mL/day may be an alternative to ≤ 0.5 mL/ (kg/h) for 6 hours. For those with only one test of creatinine, we made diagnosis of AKI with reference to the criteria of urine volume and clinical diagnosis.”

• Statistics:
o Explain the rationale for the reciprocal transformation of creatinine, including supporting normality tests.

**Reply:** To make full use of the data of serum creatinine at onset and discharge and reflect the severity of the kidney injury, we focused on the highest value of serum creatinine during the disease.

The highest value of serum creatinine itself and the values through logarithmic transformation or square root transformation were all non-abnormally distributed. But the reciprocal of it (1/ the value) is normally distributed. So, we used the reciprocal of the highest value of serum creatinine during the disease as the dependent variable. Also, the reciprocal conversion caused the residual creatinine concentration to be normally distributed after regression.

1) The normality test results of the highest serum creatinine during the disease itself and its converted values.

|  | **Kolmogorov-Smirnov (K-S)** ^a^ | | | | **Shapiro-Wilk** | |
| --- | --- | --- | --- | --- | --- | --- |
|  | **Statistic** | **df** | **Significance** | **Statistic** | **df** | **Significance** |
| **Scr_highest** | 0.349 | 65 | <0.001 | 0.273 | 65 | <0.001 |
| **lnScr_highest** | 0.154 | 65 | 0.001 | 0.762 | 65 | <0.001 |
| **SquartScr_highest** | 0.247 | 65 | <0.001 | 0.486 | 65 | <0.001 |
| **Reciprocal_Scr_highest** | 0.058 | 65 | .200* | 0.986 | 65 | 0.651 |

* the lower limit of the significance

a **Lilliefors Significance Correction**

2) **Normality test result of the unstandardized residual of the reciprocal of the highest serum creatinine during the disease after the multivariate regression analysis (model 3).**

**Kolmogorov-Smirnov (K-S) ^a^	Shapiro-Wilk**

**Statistic	df	Significance	Statistic	df Significance**

**Unstandardized Residual** 0.103; 41;	.200*	0.960; 41; .160

* the lower limit of the significance

a **Lilliefors Significance Correction**

3) The following were the normality test results of the unstandardized residual after the regression (Model 1 and 2):

**Model 1:**

**Kolmogorov-Smirnov (K-S) ^a^	Shapiro-Wilk**

**Statistic	df	Significance	Statistic df Significance**

**Unstandardized Residual**	0.107	59	0.090	0.972	59	0.195

a **Lilliefors Significance Correction**

**Model 2:**

**Kolmogorov-Smirnov (K-S) ^a^	Shapiro-Wilk**

**Statistic	df	Significance	Statistic df Significance**

**Unstandardized Residual** 0.094	59	.200*	0.977	59	0.323

* the lower limit of the significance

a **Lilliefors Significance Correction**

o Justify variable selection in multivariate models (e.g., why NLR was excluded despite univariate significance).
**Reply:** As you see, since there was statistically significant difference in NLR between AKI and non-AKI group, NLR was included in the multivariate regression analysis (Model 3) despite no significant relationship with the highest creatinine by spearman analysis (S3 table).

Variable in multivariate models included all the variables with significant relationship with the highest serum creatinine concentration during the illness by spearman two-variate relationship analysis excluding those with possible collinearity. It includes the following variables: sex, average hemoglobin, serum uric acid, blood myoglobin, serum phosphate, fibrinogen-albumin ratio. Since we can analyze 13-14 variables at most, we added those variables with significant difference between AKI and non-AKI group including SBP, DBP, heart beats per minute, length of stay, proteinuria positive or not, NLR, lymphocyte percentage, lowest urine output, serum urea. The latter three variables may be collinear with NLR or creatinine and were excluded. Total bilirubin was nearly statistically different between the two groups (P=0.056) and analyzed in the model. Age was one of the commonest demographic characteristics and added in the analysis too.

These contents were added in the statistics section.

o Report variance inflation factors to assess multicollinearity.

**Reply:** The value of VIF for FAR was 1 in Model 1 and the values of VIF for all variables in model 2 were from 1.037 to 2.421, while the VIF values for all variables in model 3 were from 1.001 to 3.484 as the following table. It suggests that the variables in the models were sufficiently independent of one another, thereby ensuring the reliability and validity of the estimated regression coefficients and associated statistical tests.

So, we added the values of variance inflation factors in the results at the end of the paragraph about the multivariate linear regression analysis: “The variance inflation factor values for all variables across the models were consistently below the commonly - accepted threshold of 10, which strongly suggests the absence of severe multicollinearity among the predictor variables.”

| Coefficient, ^a^ |  | Collinearity statistics | |
| --- | --- | --- | --- |
| Model |  | Tolerance | VIF |
| 1 | (Constant) |  |  |
|  | Sex (1, male; 0, female) | 0.46 | 2.174 |
|  | Age, years | 0.541 | 1.85 |
|  | Length of stay, days | 0.625 | 1.601 |
|  | Serum uric acid multiplied by 0.01, µmol/L | 0.528 | 1.894 |
|  | Serum phosphate, mmol/L | 0.392 | 2.553 |
|  | Blood myoglobin, ng/mL | 0.577 | 1.732 |
|  | Proteinuria or not (1, suspected or positive; 0, negative or untested) | 0.641 | 1.56 |
|  | Systolic blood pressure, mmHg | 0.275 | 3.642 |
|  | Diastolic blood pressure, mmhg | 0.31 | 3.225 |
|  | Average total bilirubin, mmol/L | 0.822 | 1.217 |
|  | Average hemoglobin, g/L | 0.583 | 1.716 |
|  | Average heart rate, beats per minute | 0.7 | 1.429 |
|  | Neutrophil /lymphocyte ratio | 0.593 | 1.687 |
|  | FAR | 0.594 | 1.684 |
| … |  |  |  |
| … |  |  |  |
| 12 | (Constant) |  |  |
|  | Serum uric acid multiplied by 0.01, µmol/L | 0.999 | 1.001 |
|  | Serum phosphate, mmol/L | 0.944 | 1.059 |
|  | FAR | 0.943 | 1.060 |
| ^a^, Dependent: reciprocal_scr_highest | | | |

4. Results
• Tables:
o Resolve discrepancies between text and tables (e.g., proteinuria cases: 17/59 in text vs. 15/59 in Table 1).

**Reply**: Thank you for your carefulness. I have corrected the error (from 17 to 15) and checked all the figures in the manuscript.

o Add interquartile ranges for median values in Table 1 and confidence intervals for beta coefficients in Table 2.

**Reply**: Thank you for your advice. In some magazines, interquartiles may be substituted by 25%, 75% quartiles. But what you said is more precise. We revised the title from “interquartile ranges” to “25% and 75% quartiles”. In table 1, the 25% and 75% quartiles have been written for non-normally distributed variables. The confidence intervals for beta coefficients existed in Table 2.

o Streamline redundant data (e.g., merge Table 1 and Supplementary Tables).

**Reply:** OK. We merged the two tables to one.

• Clinical interpretation: Translate the FAR coefficient (−0.078) into clinically meaningful terms (e.g., "A 1-unit increase in FAR corresponds to a X% increase in creatinine").

**Reply**: We revised the relative content at the first paragraph in discussion section from every 1 unit increase to 1% (0.01) increase in order to better fit the value of FAR in this study. --- “A 1%-unit increase in FAR corresponds to a 0.078% decrease in the reciprocal of the highest SCr concentration, indicating that the serum creatinine levels were concomitantly elevated.”

Supposing the uric acid concentration was 302.9umol/L and the serum phosphate level was 1.06mmol/L, the mean values in this study, we calculated the highest serum creatinine concentration with the formula in the manuscript. From which we can see the highest serum creatinine concentration increases with FAR.

As those in page 10, we have prepared a table to illustrate the clinical interpretation due to the complexity. Please refer to Page 10 for more information.

• Bias: Discuss the high rate of intentional self-poisoning (60%) and its potential impact on generalizability.

**Reply**: At first, we have described at the beginning of the method section how these patients were enrolled. They were admitted to the department of nephrology because of poisoning. So, 60% is just a proportion in this group of patients, and it’s not a figure representing the prevalence rate in the epidemiological sense.

Second, we analyzed the characteristics of this group as you mentioned. Pesticides (38.5%, n= 15) and antipsychotic sedatives (35.9%, n = 14) are the main types of poison in the patients with intentional self- poisoning (n = 39). The study cohort comprised 48.7% (n = 19) participants without pre-existing medical conditions, while those with chronic multimorbidity inclusive of mental health disorders constituted the second largest subgroup at 38.5% (n = 15) in the 39 patients (see the following table).

We added a sentence prior to the limitation: “The high proportion (60%) of intentional self-poisoning in this group requires more attention to psychological health and risk of exposure to pesticides and antipsychotic sedatives”.

In the results section, we added a paragraph about those self-poisoning: “Further analysis of the 39 patients with intentional self-poisoning demonstrated pesticides (38.5%, n = 15) and antipsychotic sedatives (35.9%, n = 14) were the main types of poison. Meanwhile, we found 48.7% (n = 19) among the 39 patients had no pre-existing medical conditions and 38.5% (n = 15) had chronic multimorbidity inclusive of mental health disorders.”

| Comorbidity | NA | Chronic disease including mental disorder | Infection |  | To sum |
| --- | --- | --- | --- | --- | --- |
| n (%) | 19 (48.7) | 15 (38.5) | 5 (12.8) |  | 39 (100) |
|  |  |  |  |  |  |
| Comorbidity- 2 | NA | Chronic disease | Mental disorder | Infection | To sum |
| n (%) | 19 (48.7) | 7 (17.9) | 8 (20.5) | 5 (12.8) | 39 (100) |
|  |  |  |  |  |  |
| Poison type | Antipsychotic sedatives | Other medication | Pesticides | Food | To sum |
| n (%) | 14 (35.9) | 9 (23.1) | 15 (38.5) | 1 (2.6) | 39 (100) |

5. Discussion
• Mechanisms: Elaborate on how FAR might mediate toxic AKI (e.g., endothelial dysfunction, microvascular thrombosis). Compare FAR’s performance to established biomarkers (e.g., NGAL, KIM-1).

**Reply**: Thanks for your advice. We have elaborated on how FAR might mediate toxic AKI and added the contents about microvascular thrombosis. Meanwhile, we have changed the relevant content to be displayed in bold.

As for the comparison of FAR with KIM-1 etc, we added some literature about the comparison of these biomarkers.

“FAR is easier to obtain than other indicators of kidney injury such as kidney injury molecular-1 (KIM-1) or neutrophil gelatinase-associated lipocalin (NGAL) etc. As biomarkers of tubular injury, KIM-1 etc are released from the proximal tubule while NGAL is released from the distal tubule [16]. Although NGAL/KIM-1 has high specificity and may reflect location of renal injury [17, 18], they are not routine tests in clinical practice. So, additional detection is required and the cost is relatively high. Moreover, false positives of NGAL may occur in sepsis-related AKI [19], while the baseline level of KIM-1 is elevated in chronic kidney disease [20]. FAR integrates the dual pathways of inflammation and coagulation, which may more comprehensively reflect the systemic pathophysiological background of AKI. FAR may be interfered by non-renal factors (such as liver cirrhosis and malnutrition), and it needs to be interpreted in combination with the clinical background [21].”

• NLR discrepancy: Address why NLR was non-significant despite prior literature (e.g., plateau-specific effects, small sample size).

**Reply**: Thank you. We added the explanation at the end of the paragraph about NLR:

“NLR was mainly affected by sepsis, acute phase of infection, or hematological disease, which is not manifest in this group (only 8 patients with acute infection among the 65 cases). The plateau-specific effects or small sample size factor may have some influence, too.”

• Limitations:
o Emphasize the small AKI subgroup (n=12) and risk of overfitting.

**Reply**: Thank you for your advice. We revised as the following:

“Second, it is worth noticing that these findings should be interpreted with caution due to **our relatively small sample size which may have risk of overfitting** and other unidentified variables such as data about hypoxia or duration of high-altitude residence prior to assessment”

o Discuss unmeasured confounders (e.g., toxin dose, comorbidities).

**Reply:** Toxin dose and comorbidities had been described in the 6^th^, 8^th^ paragraphs respectively in the results part. Some patients didn’t have the data, we added the limitation as the following:

“Second, potential biases may be introduced by missing data in the laboratory results, toxin dose and comorbidity etc.”

As for the other unmeasured confounders we made some revision as the following:

“Third, it is worth noticing that these findings should be interpreted with caution due to our relatively small sample size which may have risk of overfitting and **other unidentified variables such as data about hypoxia or duration of high-altitude residence prior to assessment**”.

6. Conclusions
• Actionable steps: Propose prospective validation of FAR in larger cohorts or explore therapeutic implications (e.g., targeting fibrinogen/albumin pathways).

**Reply**: Thanks.

Minor Comments
• Tables/Figures:
o Add a scatterplot of FAR vs. creatinine to visualize the relationship.

Reply: Thanks for your advice. The scatter plot has been added.

Fig 1, Scatter plot of FAR vs reciprocal of the highest serum creatinine during the disease.

o Standardize formatting (e.g., "n (%)", spaces around "±").

**Reply:** Thanks. We have standardized formatting as requested.

• Language:
o Correct British spellings (e.g., "analyse" → "analyze").

Reply: We have revised the manuscript as advised.

o Define abbreviations at first use (e.g., KDIGO).

Reply: We have revised the manuscript as advised.

This study addresses an important clinical question with potential implications for AKI management in high-altitude populations, but requires major revision to address the methodological and analytical issues noted above. With these improvements, your manuscript could make a valuable contribution to the literature on AKI biomarkers. I would be pleased to review a revised version that addresses these concerns.

**Reply**: Thanks for your instructions.

6. PLOS authors have the option to publish the peer review history of their article. If published, this will include your full peer review and any attached files.


If you choose “no”, your identity will remain anonymous but your review may still be made public.


**Do you want your identity to be public for this peer review?** For information about this choice, including consent withdrawal, please see our [Privacy Policy](https://track.editorialmanager.com/CL0/https:%2F%2Fwww.plos.org%2Fprivacy-policy/1/010f01961ae2ba98-346298be-1c6c-4362-8ecc-cf9c30c61804-000000/eS5vySi02H4MbpVaAJFLYu7l7oYSBHTb6IYJMJuw0c4=206).

Reviewer #1: No

Reviewer #2: **Yes:** Bardia Karim

[NOTE: If reviewer comments were submitted as an attachment file, they will be attached to this email and accessible via the submission site. Please log into your account, locate the manuscript record, and check for the action link "View Attachments". If this link does not appear, there are no attachment files.]

While revising your submission, please upload your figure files to the Preflight Analysis and Conversion Engine (PACE) digital diagnostic tool, [https://pacev2.apexcovantage.com/](https://track.editorialmanager.com/CL0/https:%2F%2Fpacev2.apexcovantage.com%2F/1/010f01961ae2ba98-346298be-1c6c-4362-8ecc-cf9c30c61804-000000/EZ9Rase8IAx8XSp2tyRUGShMZ0OInHkz8qL4KDS8oRk=206). PACE helps ensure that figures meet PLOS requirements. To use PACE, you must first register as a user. Registration is free. Then, login and navigate to the UPLOAD tab, where you will find detailed instructions on how to use the tool. If you encounter any issues or have any questions when using PACE, please email PLOS at [figures@plos.org](mailto:figures@plos.org). Please note that Supporting Information files do not need this step.
