# Supplementary material for: Higher fibrinogen-to-albumin ratio is associated with the severity of toxin associated acute kidney injury in high-altitude (3650 m) population: A retrospective analysis
Source: PLoS One. 2026 Jan 12;21(1):e0330776. doi: 10.1371/journal.pone.0330776 (PMC12795372; doi:10.1371/journal.pone.0330776)
Supplement: Attachment — Submitted filename: response_to_reviewers_auresp_2.docx [file pone.0330776.s005.docx]

PONE-D-24-26186R1

Higher fibrinogen-to-albumin ratio predicts the severity of toxin associated acute kidney injury in high-altitude (3650 m) population—a retrospective analysis

PLOS ONE

Dear Dr. Yang,

Thank you for submitting your manuscript to PLOS ONE. After careful consideration, we feel that it has merit but does not fully meet PLOS ONE’s publication criteria as it currently stands. Therefore, we invite you to submit a revised version of the manuscript that addresses the points raised during the review process.

Please submit your revised manuscript by Aug 28 2025 11:59PM. If you will need more time than this to complete your revisions, please reply to this message or contact the journal office at [plosone@plos.org](mailto:plosone@plos.org). When you're ready to submit your revision, log on to https://www.editorialmanager.com/pone/ and select the 'Submissions Needing Revision' folder to locate your manuscript file.

Please include the following items when submitting your revised manuscript:

- A rebuttal letter that responds to each point raised by the academic editor and reviewer(s). You should upload this letter as a separate file labeled 'Response to Reviewers'.
- A marked-up copy of your manuscript that highlights changes made to the original version. You should upload this as a separate file labeled 'Revised Manuscript with Track Changes'.
- An unmarked version of your revised paper without tracked changes. You should upload this as a separate file labeled 'Manuscript'.

If you would like to make changes to your financial disclosure, please include your updated statement in your cover letter. Guidelines for resubmitting your figure files are available below the reviewer comments at the end of this letter.

If applicable, we recommend that you deposit your laboratory protocols in protocols.io to enhance the reproducibility of your results. Protocols.io assigns your protocol its own identifier (DOI) so that it can be cited independently in the future. For instructions see: [https://journals.plos.org/plosone/s/submission-guidelines#loc-laboratory-protocols](https://track.editorialmanager.com/CL0/https:%2F%2Fjournals.plos.org%2Fplosone%2Fs%2Fsubmission-guidelines%23loc-laboratory-protocols/1/010f01980b2019a9-254a05b5-307b-4dbe-81d1-fe81cac6b527-000000/hrV1BBpx_gRdYDlOdwoD-7ZLFNy4WrgAO_0NlZUJrlA=219). Additionally, PLOS ONE offers an option for publishing peer-reviewed Lab Protocol articles, which describe protocols hosted on protocols.io. Read more information on sharing protocols at [https://plos.org/protocols?utm_medium=editorial-email&utm_source=authorletters&utm_campaign=protocols](https://track.editorialmanager.com/CL0/https:%2F%2Fplos.org%2Fprotocols%3Futm_medium=editorial-email%26utm_source=authorletters%26utm_campaign=protocols/1/010f01980b2019a9-254a05b5-307b-4dbe-81d1-fe81cac6b527-000000/CkQCK6B2YuTrPeyR7kXY6iBK5xMdGVjGO0aU795mdAA=219).

We look forward to receiving your revised manuscript.

Kind regards,

Mohammad Barary, MD
Academic Editor
PLOS ONE

**Journal Requirements:**

If the reviewer comments include a recommendation to cite specific previously published works, please review and evaluate these publications to determine whether they are relevant and should be cited. There is no requirement to cite these works unless the editor has indicated otherwise. 


[Note: HTML markup is below. Please do not edit.]

Reviewers' comments:

Reviewer's Responses to Questions

**Comments to the Author**

1. If the authors have adequately addressed your comments raised in a previous round of review and you feel that this manuscript is now acceptable for publication, you may indicate that here to bypass the “Comments to the Author” section, enter your conflict of interest statement in the “Confidential to Editor” section, and submit your "Accept" recommendation.

Reviewer #3: All comments have been addressed

Reviewer #4: All comments have been addressed

Reply: Thank for your guidance.

2. Is the manuscript technically sound, and do the data support the conclusions?

The manuscript must describe a technically sound piece of scientific research with data that supports the conclusions. Experiments must have been conducted rigorously, with appropriate controls, replication, and sample sizes. The conclusions must be drawn appropriately based on the data presented.

Reviewer #3: Partly

Reviewer #4: Yes

Reply: Thank for your guidance.

3. Has the statistical analysis been performed appropriately and rigorously?

Reviewer #3: Yes

Reviewer #4: Yes

Reply: Thank for your guidance.

4. Have the authors made all data underlying the findings in their manuscript fully available?

The [PLOS Data policy](http://track.editorialmanager.com/CL0/http:%2F%2Fwww.plosone.org%2Fstatic%2Fpolicies.action%23sharing/1/010f01980b2019a9-254a05b5-307b-4dbe-81d1-fe81cac6b527-000000/7a_FDK8q1U5T80WEyXsNqw3DehF-P9KNIHNdGbKD7_0=219) requires authors to make all data underlying the findings described in their manuscript fully available without restriction, with rare exception (please refer to the Data Availability Statement in the manuscript PDF file). The data should be provided as part of the manuscript or its supporting information, or deposited to a public repository. For example, in addition to summary statistics, the data points behind means, medians and variance measures should be available. If there are restrictions on publicly sharing data—e.g. participant privacy or use of data from a third party—those must be specified.

Reviewer #3: Yes

Reviewer #4: Yes

Reply: Thank for your guidance.

5. Is the manuscript presented in an intelligible fashion and written in standard English?

PLOS ONE does not copyedit accepted manuscripts, so the language in submitted articles must be clear, correct, and unambiguous. Any typographical or grammatical errors should be corrected at revision, so please note any specific errors here.

Reviewer #3: Yes

Reviewer #4: Yes

Reply: Thank for your guidance.

6. Review Comments to the Author

Please use the space provided to explain your answers to the questions above. You may also include additional comments for the author, including concerns about dual publication, research ethics, or publication ethics. (Please upload your review as an attachment if it exceeds 20,000 characters)

**Reviewer #3:** Study Design Clarification:

Specify the study type (cross-sectional, retrospective, etc.).
Reply: Thank for your instruction. It is a retrospective study noted in the title, methods and at the second paragraph in the introduction section of the paper.

Provide detailed inclusion and exclusion criteria.

Reply: Thank for your instruction. The inclusion and exclusion criteria have been clearly demonstrated at the beginning of the methods. However, as your requirement, we modified it as the following:

“Clinical data of poisoned inpatients fulfilling the criterion were retrospectively analyzed during July 20th 2022 to July 20th 2023 to determine the clinical characteristics and risk factors of renal damage in the plateau area…

Inclusion criterion: All poisoned patients who had been hospitalized **consecutively** to our wards and had electronic medical records available in the past ten years were enrolled.

Exclusion criterion: The patients who did not have urine volume record and renal function data during the illness were excluded.”

Was the sampling random, consecutive, or convenience-based?

Reply: Thank for your instruction. It is consecutive, which was added into the inclusion criterion in red as above.

Statistical Detail:

Include confidence intervals and effect sizes for associations.

Reply: Thank for your instruction. The confidence intervals and effect sizes have been listed in table 2 for associations. In multivariable linear regression analysis, the absolute value of normalized coefficient---beta value was the effect size which can compare the devotion of different variables to the independent variable (the reciprocal of the highest serum creatinine abbreviated as SCr in this study). Positive value indicates the value of independent variable (the reciprocal of the highest scr in this study) will increase with the increase of this variable, while negative indicates protective or decreasing the value of independent variable (the reciprocal of the highest scr). Ex. In model 3, the effect sizes of FAR, uric acid and phosphate are close, and serum uric acid devotes a little more to the reciprocal of SCr than the other two variables (-0.424 to -0.400, -0.399 respectively).

Consider multivariate logistic regression to control for confounders (e.g., age, gender, diagnosis).

Reply: Thank for your instruction. There were only 12 cases diagnosed as AKI. Due to the limited number of events, we can only analyze the influence of 1 to 3 confounders or variables (12 divided by 5 to 10) on the dependent variable. Moreover, some confounders have missing values, then the analyzed cases in the multivariate logistic regression will be less. Taking model 3 as an example, the different variables may have missing values in different patient, then only 23 cases were analyzed in logistic regression. So, it needs more cases in the study to utilize multivariate logistic regression. However, the highest serum creatinine is a continuous variable and all 65 patients had the value. In multivariate linear regression, it allowed pairwise analysis for confounders with missing value. Since logistic regression uses a categorical dependent variable (AKI or not) which will lose some information than the quantitative variable (the reciprocal of the highest serum creatinine) in linear regression in this study. So, it is not advisable. In the multivariate linear regression, FAR has been adjusted by sex, age etc.

We added a sentence in the paragraph before Table 2 as the following. “It demonstrated that FAR was associated negatively with the reciprocal of highest serum creatinine, even after adjustment by other demographic or laboratory variables (Table 2).”

Clearly describe how the chi-square test was applied and its assumptions validated.

Reply: Thank for your instruction. We have revised as the following. “Categorical variables are compared with chi-square tests, corrected for continuity if it is a 2* 2 table or Fisher’s exact test if >20% of cells have the expected count < 5 in a table larger than 2×2.”

Presentation of Results:

Improve clarity of tables (use consistent decimal formatting, label variables clearly).
Reply: Thank for your instruction. We have revised the tables as request.

Provide total sample size in each analysis for transparency.
Reply: Thank for your instruction. We have labeled the sample size in yellow in tables.

For multivariate linear regression, I listed the relative part below (/missing pairwise, different from multivariate logistic regression which allowed only listwise for missing values):

Model 1:

|  | n |
| --- | --- |
| reciprocal of scr_highest | 65 |
| FAR | 59 |

Model 2:

|  | n |
| --- | --- |
| reciprocal of scr_highest | 65 |
| sex: male,1; female:0 | 65 |
| Age, years | 65 |
| Length of stay, days | 65 |
| SBP at onset, mmHg | 64 |
| DBP at onset, mmHg | 64 |
| HR_average | 65 |
| FAR | 59 |

Model 3:

|  | n |
| --- | --- |
| reciprocal of scr_highest | 65 |
| sex: male,1; female:0 | 65 |
| Age, years | 65 |
| Length of stay, days | 65 |
| SBP at onset, mmHg | 64 |
| DBP at onset, mmHg | 64 |
| HR_average | 65 |
| FAR | 59 |
| Serum phosphate, mmol/L | 55 |
| Blood myoglobin, ng/mL | 41 |
| NLR | 58 |
| Uric acid*0.01 | 44 |
| Proteinuria or not | 65 |
| Average hemoglobin, g/L | 63 |
| Total bilirubin, mmol/L | 62 |

Strengthen the interpretation of results with comparisons to other Indian and international studies.

Reply: We appreciate this constructive suggestion. To address this, the Discussion section has been expanded to include comparative analyses with multiple international studies, including:

• Indian research (Ref. 19)

• Multicenter collaborations (Ref. 12: Italy/India)

• Studies from Japan (Ref. 31), Australia (Ref. 29), France (Ref. 6), the United States (Refs. 2, 14, 36), and Brazil (Ref. 1).

Additionally, we conducted a systematic PubMed search for recent Indian literature relevant to our study domain. After critical evaluation of the following publications, we determined that they do not contribute substantively to the interpretation of our results:

1. Parthasarathy et al. (2021, Nephrology)

Parthasarathy R., Mathew M., Koshy P., et al. Traditional medicines prescribed for prevention of COVID-19: Use with caution. Nephrology (Carlton) 2021, 26(12): 961-964

Rationale for exclusion: Reports an isolated case of nephrotoxicity linked to Indian traditional COVID-19 prophylactics (kabasura/nilavembu kudineer), which does not align with our mechanistic focus.

2. Ralph et al. (2024, Toxicon)

Ralph R., Sharma D., Jain R., e al. Protobothrops jerdonii (Jerdon's pit viper) and Protobothrops himalayanus (Himalayan lance-headed pit viper) bites: Clinical report on envenomings from North-East India, managed through remote consultation by a national-level Poison control center. Toxicon 2024, 242:107704

Rationale for exclusion: Documents acute kidney injury (AKI) from viper envenomation—a contextually distinct etiology from our research parameters.

3. Pannu (2019, Current Drug Metabolism)

Pannu K. Methotrexate overdose in clinical practice. Curr Drug Metab 2019,20(9): 714-719

Rationale for exclusion: Focuses on methotrexate overdose toxicology, beyond our study scope.

4. Sirur et al. (2022, Wilderness & Environmental Medicine)

Sirur F.M., Balakrishnan J.M. and Lath V.. Hump-Nosed Pit Viper Envenomation in Western Coastal India: A Case Series. Wilderness Environ Med 2022, 33(4): 399-405

Rationale for exclusion: Examines hump-nosed viper envenomation syndromes in Western India, unrelated to our pathophysiological framework.

5. Renu et al. (2022, Molecules)

[Renu](https://pubmed.ncbi.nlm.nih.gov/?term=Renu+K&cauthor_id=36080424) K,  [Mukherjee](https://pubmed.ncbi.nlm.nih.gov/?term=Mukherjee+AG&cauthor_id=36080424) AG, [Wanjari](https://pubmed.ncbi.nlm.nih.gov/?term=Wanjari+UR&cauthor_id=36080424)  UR, et al. Misuse of Cardiac Lipid upon Exposure to Toxic Trace Elements-A Focused Review. Molecules, 2022;27(17):5657. doi: 10.3390/molecules27175657.

Rationale for exclusion: Explores trace element-induced cardiotoxicity through lipid dysregulation, diverging from our core investigative pathways.

We confirm that all citations incorporated in the revised manuscript directly substantiate our comparative analysis. Thank you for your guidance throughout this revision process.

Expand on public health implications.

Reply: Thank for your instruction. I highlighted the content in page 19: “The high proportion (60%) of intentional self-poisoning in this group requires more attention to psychological health and risk of exposure to pesticides and antipsychotic sedatives.” And the first sentence in the conclusion was about the public implications “The public needs to be well informed to minimize the exposure to medicine, pesticide, mushroom etc”.

As requested, we expanded the contents as the following.

“Routine mental health and suicide risk screening in primary care and relevant community settings were recommended for patients residing at high altitude areas. For high self- harm risk population, appropriate mental health services and supervision should be strengthened in caregiver and family. Second, pesticide and antipsychotic sedative access requires tighter control and monitoring. Both the sales end and the client end need to strengthen regulations on sales, distribution, packaging, safe storage (locked boxes), and to monitor unusual dispensing patterns.”

Acknowledge limitations such as potential selection bias and self-reporting of tobacco use.

Reply: Thank for your instruction. As request, we acknowledge the limitations at the end of discussion section: “Despite adjustment for potential confounding factors, potential selection bias and residual bias might still exist.” Sorry, tobacco use is outside our focus in this study.

**Reviewer #4:** Thank you for making all the recommended changes, this is an interesting topic in this small population.

Reply: Thank for your instruction.

7. PLOS authors have the option to publish the peer review history of their article ([what does this mean?](https://track.editorialmanager.com/CL0/https:%2F%2Fjournals.plos.org%2Fplosone%2Fs%2Feditorial-and-peer-review-process%23loc-peer-review-history/1/010f01980b2019a9-254a05b5-307b-4dbe-81d1-fe81cac6b527-000000/lYYvKG3fMVx9YWI7vy2hJ5I7KYJBhCmB2LCFADF1oXA=219)). If published, this will include your full peer review and any attached files.


If you choose “no”, your identity will remain anonymous but your review may still be made public.


**Do you want your identity to be public for this peer review?** For information about this choice, including consent withdrawal, please see our [Privacy Policy](https://track.editorialmanager.com/CL0/https:%2F%2Fwww.plos.org%2Fprivacy-policy/1/010f01980b2019a9-254a05b5-307b-4dbe-81d1-fe81cac6b527-000000/mvHyvPjQWp6TpenziYpT2hQZZzawjLbYiHlPQERQoAo=219).

Reviewer #3: No

Reviewer #4: No

 Reply: Thank for your instruction.

[NOTE: If reviewer comments were submitted as an attachment file, they will be attached to this email and accessible via the submission site. Please log into your account, locate the manuscript record, and check for the action link "View Attachments". If this link does not appear, there are no attachment files.]

While revising your submission, please upload your figure files to the Preflight Analysis and Conversion Engine (PACE) digital diagnostic tool, [https://pacev2.apexcovantage.com/](https://track.editorialmanager.com/CL0/https:%2F%2Fpacev2.apexcovantage.com%2F/1/010f01980b2019a9-254a05b5-307b-4dbe-81d1-fe81cac6b527-000000/yu68-EHL_GctjgCIyD-5t2zfT00uUiwY3dCVE26m9UQ=219). PACE helps ensure that figures meet PLOS requirements. To use PACE, you must first register as a user. Registration is free. Then, login and navigate to the UPLOAD tab, where you will find detailed instructions on how to use the tool. If you encounter any issues or have any questions when using PACE, please email PLOS at [figures@plos.org](mailto:figures@plos.org). Please note that Supporting Information files do not need this step.
