# Supplementary material for: Higher fibrinogen-to-albumin ratio is associated with the severity of toxin associated acute kidney injury in high-altitude (3650 m) population: A retrospective analysis
Source: PLoS One. 2026 Jan 12;21(1):e0330776. doi: 10.1371/journal.pone.0330776 (PMC12795372; doi:10.1371/journal.pone.0330776)
Supplement: Attachment — Submitted filename: Response_to_Reviewers_auresp_3.docx [file pone.0330776.s006.docx]

PONE-D-24-26186R2 Higher fibrinogen-to-albumin ratio predicts the severity of toxin associated acute kidney injury in high-altitude (3650 m) population—a retrospective analysis PLOS ONE

Dear Dr. Yang,

Thank you for submitting your manuscript to PLOS ONE. After careful consideration, we feel that it has merit but does not fully meet PLOS ONE’s publication criteria as it currently stands. Therefore, we invite you to submit a revised version of the manuscript that addresses the points raised during the review process.

Please submit your revised manuscript by Dec 28 2025 11:59PM. If you will need more time than this to complete your revisions, please reply to this message or contact the journal office at [plosone@plos.org](mailto:plosone@plos.org). When you're ready to submit your revision, log on to <https://www.editorialmanager.com/pone/> and select the 'Submissions Needing Revision' folder to locate your manuscript file.

Please include the following items when submitting your revised manuscript:

- A rebuttal letter that responds to each point raised by the academic editor and reviewer(s). You should upload this letter as a separate file labeled 'Response to Reviewers'.
- A marked-up copy of your manuscript that highlights changes made to the original version. You should upload this as a separate file labeled 'Revised Manuscript with Track Changes'.
- An unmarked version of your revised paper without tracked changes. You should upload this as a separate file labeled 'Manuscript'.

REPLY: Thanks. We have completed the revision and uploaded.

If you would like to make changes to your financial disclosure, please include your updated statement in your cover letter. Guidelines for resubmitting your figure files are available below the reviewer comments at the end of this letter.

REPLY: Thanks. No change.

If applicable, we recommend that you deposit your laboratory protocols in protocols.io to enhance the reproducibility of your results. Protocols.io assigns your protocol its own identifier (DOI) so that it can be cited independently in the future. For instructions see: <https://journals.plos.org/plosone/s/submission-guidelines#loc-laboratory-protocols>. Additionally, PLOS ONE offers an option for publishing peer-reviewed Lab Protocol articles, which describe protocols hosted on protocols.io. Read more information on sharing protocols at <https://plos.org/protocols?utm_medium=editorial-email&utm_source=authorletters&utm_campaign=protocols>.

REPLY: Thanks. It is not applicable.

We look forward to receiving your revised manuscript.

Kind regards,

Shivkumar Gopalakrishnan, MD

Academic Editor PLOS ONE

Journal Requirements:

If the reviewer comments include a recommendation to cite specific previously published works, please review and evaluate these publications to determine whether they are relevant and should be cited. There is no requirement to cite these works unless the editor has indicated otherwise.

Please review your reference list to ensure that it is complete and correct. If you have cited papers that have been retracted, please include the rationale for doing so in the manuscript text, or remove these references and replace them with relevant current references. Any changes to the reference list should be mentioned in the rebuttal letter that accompanies your revised manuscript. If you need to cite a retracted article, indicate the article’s retracted status in the References list and also include a citation and full reference for the retraction notice.

REPLY: Thanks. All cited papers have been checked.

[Note: HTML markup is below. Please do not edit.]

Reviewers' comments:

Reviewer's Responses to Questions

Comments to the Author

1. If the authors have adequately addressed your comments raised in a previous round of review and you feel that this manuscript is now acceptable for publication, you may indicate that here to bypass the “Comments to the Author” section, enter your conflict of interest statement in the “Confidential to Editor” section, and submit your "Accept" recommendation.

Reviewer #4: (No Response)

Reviewer #5: All comments have been addressed

REPLY: Thanks.

2. Is the manuscript technically sound, and do the data support the conclusions?

The manuscript must describe a technically sound piece of scientific research with data that supports the conclusions. Experiments must have been conducted rigorously, with appropriate controls, replication, and sample sizes. The conclusions must be drawn appropriately based on the data presented.

Reviewer #4: Partly

Reviewer #5: Yes

REPLY: Thanks.

3. Has the statistical analysis been performed appropriately and rigorously?

Reviewer #4: Yes

Reviewer #5: Yes

REPLY: Thanks.

4. Have the authors made all data underlying the findings in their manuscript fully available?

The PLOS Data policy requires authors to make all data underlying the findings described in their manuscript fully available without restriction, with rare exception (please refer to the Data Availability Statement in the manuscript PDF file). The data should be provided as part of the manuscript or its supporting information, or deposited to a public repository. For example, in addition to summary statistics, the data points behind means, medians and variance measures should be available. If there are restrictions on publicly sharing data—e.g. participant privacy or use of data from a third party—those must be specified.

Reviewer #4: Yes

Reviewer #5: Yes

REPLY: Thanks.

5. Is the manuscript presented in an intelligible fashion and written in standard English?

PLOS ONE does not copyedit accepted manuscripts, so the language in submitted articles must be clear, correct, and unambiguous. Any typographical or grammatical errors should be corrected at revision, so please note any specific errors here.

Reviewer #4: Yes

Reviewer #5: Yes

REPLY: Thanks.

6. Review Comments to the Author

Please use the space provided to explain your answers to the questions above. You may also include additional comments for the author, including concerns about dual publication, research ethics, or publication ethics. (Please upload your review as an attachment if it exceeds 20,000 characters)

Reviewer #4: Thank you for submitting this amended article.

I would suggest the following changes

Introduction

1. Change "food poisoning" to "ingestion of known toxic substances" (mushrooms/ fish gall cysts) may this be deliberate or accidental. Food poisoning in many countries is where a person becomes mildly/ moderately unwell often due to poor hygiene during food preparation that leads to gastrointestinal illness.

REPLY: Thanks. Except one patient, all the other 11 patients ate **poisonous mushroom, wild vegetables or contaminated fish** by mistake and they thought them edible. To distinguish with the poisoning from medicine, pesticides, we used the “food poisoning” in general, while “ingestion of known toxic substances” may include ingestion of medicine, pesticides, household **chemicals,** **poisonous mushroom, toxic seafood,** **contaminated fish etc**. So, we think changing to “toxic substances eaten as food” may be more suitable to this context.

2. On line 50 you say that AKI definition varies widely- but KDGIO international guidance has been in place since 2012. Please amend to say that prior to 2012 definitions varied, and then go on to talk about the impact that had on previous research.

REPLY: Thanks. We revised it as the following: “However, the definition of AKI varies widely across the literature prior to 2012”

Methods. 3. Within the methods section you need to state how you identified these patients.

REPLY: Thanks. We have stated it clearly in the method section:

Inclusion criterion: All poisoned patients who had been hospitalized consecutively to our wards and had electronic medical records available in the past ten years were enrolled.

Exclusion criterion: The patients who had neither urine volume record nor renal function data during the illness were excluded.

4. Why did you exclude patients who did not have their urine output recorded during their illness? How did this impact on your sample size.

REPLY: Thanks. In the method section, we have stated that the exclusion criterion was those patients who did not have urine volume record and renal function data during the illness. In the result section, we have also stated clearly that: “one patient was excluded due to lack of both renal function and urine volume record”.

If the patient has neither serum creatinine data nor urine volume data, how to judge whether they have AKI according to AKI definition? To make it more clearer, we revised the sentence as “The patients who had neither urine volume record nor renal function data during the illness were excluded.”

5. How did you ensure that AKI based on urine output was true? It is well known that urine output documentation is poor outside of critical care areas. This needs to make very clear as a limitation as 1/3 of your AKI patients may not have had an AKI at all but only poor fluid balance documentation. You also need to state how many of these patients were determined to be AKI based on urine output alone here (I know this is in your results section).

REPLY: Thanks. What you said about the limitation of urine criterion is right, we have stated how to use urine volume criterion in the method: “AKI status was defined as literature [8]. An increase in serum creatinine (SCr) concentration from baseline can be substituted as a decrease from the baseline during follow-up if there are no prior tests. A urine output less than 400 mL/day may be an alternative to ≤ 0.5 mL/ (kg/h) for 6 hours. For those with only one test of serum creatinine, we made diagnosis of AKI with reference to the criteria of urine volume and clinical diagnosis.”

We also worry about the confusion of urine volume criterion for AKI, so we analyzed those defined using serum creatinine criterion only. “If we defined AKI without regard to urine output, then only 8 patients had AKI (Table 1 in S1 Tables).” Four patients who were defined as AKI according to urine volume criterion had urine volume of 170-300ml/d at admission and 1900-4850ml/d at discharge after therapy. They had suffered from confusion (n = 3) or drowsiness (n=1). All the four patients maybe had only poor fluid balance after poisoning, but if no therapy was commenced, more serious kidney injury may happen.

Results This section is quite muddled with the results being focused on the poisoning elements (which is important but needs to be presented more clearly).

REPLY: Thanks. All the 65 patients were admitted to wards because of poisoning due to different poisons at plateau, so poisoning elements may be different in some degree from those in plain areas. If there is any content not stated clearly, please don’t hesitate to tell us and we will made it clearer.

6. The first part of your results should be that only 12 patients of the 65 identified were found to have AKI. You spend a long time discussing the causes of poisoning in all patients- but as this article is about AKI in this patient group your results section should begin with the most important finding.

REPLY: Thanks. Our paper focused on two points, namely toxic kidney injury using the criterion of AKI and plateau area. All analysis about poisoning elements was to see if there was any difference in plateau compared with those in plain areas.

7. Within your results it would provide more clarity if you provided how many of the 35 patients who were "poisoned" with medication were intentional overdoses.

REPLY: Thanks. Line 146 in manuscript with marks: intentional self-poisoning: n =39; intentional medical overdoses: n=23.

We revised the results as the following:

“Further analysis of the 39 patients with intentional self-poisoning demonstrated the main types of poisons were pesticides (38.5%, n = 15) and medication (59.0%, n=23), while antipsychotic sedatives (60.9%, n = 14) were the main type of medicine.”

8. Is Hemorperfusion the same as hemofiltration? Which I suspect it is- if patients are getting this as a first line treatment it may explain why patients are developing AKI (removal of toxin = no toxins to cause AKI). This should be included in your discussion.

REPLY: Thanks. Hemoperfusion is different from hemofiltration and the former was more useful in the removal of toxins. Whether removal of toxin means no or smaller quantity of toxins to cause AKI depend on the following points: 1) How long the patient was transferred to emergency after poisoning; 2) How long the blood purification was commenced after admission; 3) The protein binding capacity of the toxins. Multiple hemoperfusion therapy may be necessary for those taking toxins with high protein binding capacity.

Among the 65 patients diagnosed as poisoning, thirty-four patients (52.3%) underwent blood purification. Among them, hemoperfusion was carried out in 33 patients for 1-4 times.

Discussion 9. On line 277 you use the word etc- please clarify what else is reported to be prognostic for mortality.

REPLY: Thanks. In patients with acute glyphosate poisoning (n=73), leukocyte and coagulation time etc. were reported to be prognostic for mortality [7]. The other factors included gamma-glutamyl transpeptidase (GGT), age and medical contact time (categories: 1, within 2h; 2, 2-6h; 3, above 6h).

Conclusion 10. You have not appropriately addressed the previous reviewers comments here. More detail is required in understanding what the public needs to do. You do not want people to minize their exposure to antidepressant/ anti psychotic medications- they need to be aware of risks of toxicity of overdosing.

REPLY: Thanks. We revised the first sentence in the conclusion section as “The public needs to be well informed to minimize the chance of exposure to excessive medicine, wild vegetables, toxic mushrooms, and pesticides. Traditional Tibetan medicine, unique to this plateau region, requires professional guidance for its identification, processing, and dosage. Mental well-being in plateau areas must be prioritized, and regulatory oversight of pesticides and antipsychotic sedatives need to be strengthened to mitigate the risks of overdose and toxic exposure.”

11. Line 336 has a statement in with no clarification or depth to justify it.

REPLY: Thanks. We revised it as: “The public needs to be well informed to minimize the chance of exposure to excessive medicine, wild vegetables, toxic mushrooms, and pesticides. Traditional Tibetan medicine, unique to this plateau region, requires professional guidance for its identification, processing, and dosage. Mental well-being in plateau areas must be prioritized, and regulatory oversight of pesticides and antipsychotic sedatives need to be strengthened to mitigate the risks of overdose and toxic exposure.”

12. On line 336 you use the word etc- please clarify what else is required.

REPLY: Thanks. **Etc:** wild vegetables, **toxic seafood, contaminated fish or** **toxic substances eaten as food.** We revised it as the above reply.

You need a clear limitations section that is separate from the discussion.

REPLY: Thanks. It has been separated.

Reviewer #5: Dear Authors please provide this additional information and modifications:

Please replace all instances of “predicts” with “is associated with” throughout the manuscript, including the title and abstract, unless you add a validated prediction framework (e.g., model development with internal validation, calibration, and performance metrics).

REPLY: Thanks. All have been corrected.

Please provide the assay methods and units for fibrinogen and albumin, and state the exact formula used to compute the fibrinogen-to-albumin ratio (FAR). Clarify the measurement units employed (e.g., g/L vs. mg/dL) to ensure reproducibility and interpretability.

REPLY: Thanks. Fibrinogen is assayed by the thrombin clotting method, while albumin is measured by the bromcresol green method, both are reported in g/L.

Fibrinogen-to-albumin ratio (FAR)= Fibrinogen (g/L)/ serum albumin during the illness (g/L)

This formula was added to the note for Table 1.

Please define precisely when each laboratory value—including fibrinogen, albumin, and creatinine—was obtained relative to poisoning onset, emergency department presentation, and any treatments (e.g., hemoperfusion). Indicate whether values were collected before or after interventions and specify the time window used for analyses.

REPLY: Thanks. Fibrinogen, albumin, and creatinine values measured at emergency department before any treatments were regarded as data at onset. We added a statement in the statistical part in method section:“Those measured at emergency department before any treatments were regarded as data at onset.”

The study period is the time in ward admitted from emergency department. We are responsible for the blood purification therapy both in the emergency department and ward.

7. PLOS authors have the option to publish the peer review history of their article (what does this mean?). If published, this will include your full peer review and any attached files.

If you choose “no”, your identity will remain anonymous but your review may still be made public.

Do you want your identity to be public for this peer review? For information about this choice, including consent withdrawal, please see our Privacy Policy.

Reviewer #4: No

Reviewer #5: No

[NOTE: If reviewer comments were submitted as an attachment file, they will be attached to this email and accessible via the submission site. Please log into your account, locate the manuscript record, and check for the action link "View Attachments". If this link does not appear, there are no attachment files.]

To ensure your figures meet our technical requirements, please review our figure guidelines: <https://journals.plos.org/plosone/s/figures>

You may also use PLOS’s free figure tool, NAAS, to help you prepare publication quality figures: <https://journals.plos.org/plosone/s/figures#loc-tools-for-figure-preparation>.

NAAS will assess whether your figures meet our technical requirements by comparing each figure against our figure specifications.

REPLY: Thanks. Our figure fulfilled the requirement.
